# MEMS-Based Electrochemical Seismometer with a Sensing Unit Integrating Four Electrodes

**DOI:** 10.3390/mi12060699

**Published:** 2021-06-15

**Authors:** Wenjie Qi, Chao Xu, Bowen Liu, Xu She, Tian Liang, Deyong Chen, Junbo Wang, Jian Chen

**Affiliations:** 1 State Key Laboratory of Transducer Technology, Aerospace Information Research Institute, Chinese Academy of Sciences, Beijing 100190, China; qiwenjie16@mails.ucas.ac.cn (W.Q.); xuchao16@mails.ucas.ac.cn (C.X.); liubowen17@mails.ucas.ac.cn (B.L.); shexu18@mails.ucas.ac.cn (X.S.); liangtian18@mails.ucas.ac.cn (T.L.); chenjian@mail.ie.ac.cn (J.C.); 2 School of Electronic, Electrical and Communication Engineering, University of Chinese Academy of Sciences, Beijing 100049, China

**Keywords:** electrochemical seismometers, integrating four electrodes, sub-micron alignment accuracy, high consistency

## Abstract

This paper presents a new process to fabricate a sensing unit of electrochemical seismometers using only one silicon–glass–silicon bonded wafer. By integrating four electrodes on one silicon–glass–silicon bonded wafer, the consistency of the developed sensing unit was greatly improved, benefiting from the high alignment accuracy. Parameter designs and simulations were carried out based on this sensing unit, which indicated that the sensitivities of the developed electrochemical seismometer decreased with the decrease in the number of flow holes in the sensing unit, and the initial stabilization time decreased gradually with the decrease in the thickness of the glass layer. Based on experimental results of four devices, the peak sensitivity was quantified as 5345.45 ± 43.78 V/(m/s) at 2 Hz, which proved high consistency of the fabricated electrochemical seismometer. In terms of the responses to random ground motions, high consistencies between the developed electrochemical seismometer and the commercial counterpart of CME6011 (R-sensors, Moscow, Russia) were found, where the developed electrochemical seismometer produced comparable noise levels to those of CME6011. These results validated the performance of the device and it may function as an effective tool for a variety of applications.

## 1. Introduction

A seismometer is a sensor that detects and converts vibration signals into electrical signals, and is widely used in oil and gas resource exploration, building and bridge detection, global seismographic networks, seabed earthquake monitoring and other fields [1,2,3]. According to the working principle, seismometers can be divided into a variety of types, such as magnetoelectric moving-coil seismometers, optical fiber seismometers, capacitive seismometers, piezoelectric seismometers and electrochemical seismometers [4,5,6,7,8,9,10]. Compared with the others, electrochemical seismometers are characterized by high sensitivities, wide frequency bands, low power consumption, high shock resistance (no vulnerable parts) and large installation angles, and thus they play a key role in deep geophysical explorations and ocean-bottom seismic monitoring [11,12].

The development of electrochemical seismometers can be traced back to the 1950s. The Defense Research Laboratory of the University of Texas began to study infrasonic microphones and acoustic pressure detectors based on the principle of electrochemistry at that time, with the name of “Solion” (ions in solution) [13,14]. Significant studies on Solion’s applications in motion detection were continued in Russia, where the concept of electrochemical seismometers was introduced [15,16,17].

The sensing unit of traditional commercial electrochemical seismometers is fabricated by platinum net-waving and ceramic sinter technologies. There are many problems in this conventional manufacturing process: (1) Ceramic flakes are easy to crack, resulting in low yields; (2) electrode and insulating layers are difficult to align, resulting in poor consistencies; (3) parameters of the sensing unit are difficult to adjust, leading to limited performances [18,19].

In order to solve these problems, MEMS technologies have been introduced to manufacture sensing units of electrochemical seismometers in recent years. He et al. reported sensing units where several microfabricated layers of electrodes and insulation were stacked together, and due to poor alignment accuracies, the reported electrochemical microseismometers suffered from the key issue of low consistencies [20]. In order to reduce the challenges in manufacture and assembly, Deng et al. and Huang et al., based on different methods of fabrication, developed sensing units with integrated anodes and cathodes [21,22,23]. Nevertheless, since two chips still needed to be manually aligned and assembled, they still suffered from poor consistencies.

To further reduce the number of silicon wafers needed, Sun et al. reported sensing units with four electrodes integrated on one chip, and patterned SU-8 photoresist acted as an insulating layer between the anode and cathode [24]. However, because the area of the cathode was too small, the sensitivity of the electrochemical seismometer was greatly reduced. Furthermore, the manufacturing process of fabricating electrodes on flexible materials resulted in low yields. In addition, Zheng et al. fabricated sensing units with two pairs of electrodes integrated on both sides of one chip [25]. However, the performance of the electrochemical seismometer was poor because there were no electrodes on the side walls of flow holes. Furthermore, She et al. proposed a MEMS-based electrochemical seismometer relying on a CAC (cathode-anode-cathode) integrated three-electrode structure [26]. However, due to the differences in the working principle, the noise of the electrochemical seismometer was too high at an intermediate frequency and the linearity was poor under large vibration inputs.

To address these issues, this paper presents a MEMS-based electrochemical seismometer with a sensing unit integrating four electrodes. Benefiting from no manual alignment and enlarged electrode areas, the electrochemical seismometer developed in this study was characterized by high consistency and high sensitivity.

## 2. Structure and Working Principle

**Figure 1:**
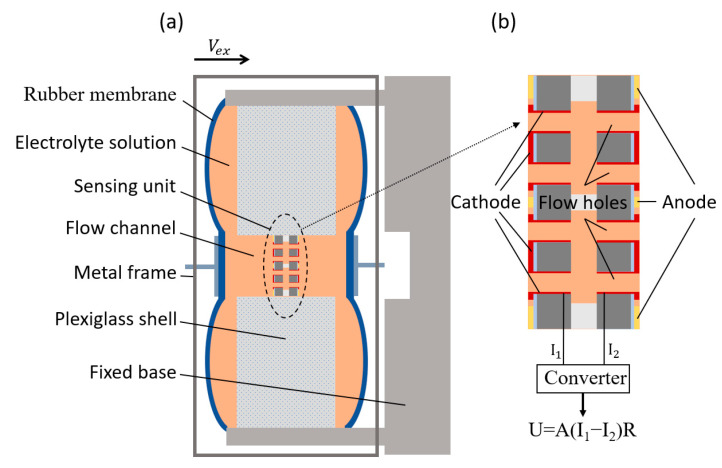
(**a**) Schematic diagram of the MEMS-based electrochemical seismometer developed in this study, consisting of: (1) two rubber membranes, (2) an electrolyte solution, (3) a sensing unit, (4) a flow channel, (5) a metal frame, (6) a plexiglass shell and (7) a fixed base. Two rubber membranes are sealed on the plexiglass block to form a sealed cavity, which is filled with the electrolyte solution, and the sensing unit is fixed in the middle of the flow channel. (**b**) Enlarged schematic diagram of the sensing unit with (8) two anodes, (9) two cathodes and (10) flow holes.

As shown in Figure 1a, the MEMS-based electrochemical seismometer developed in this study is composed of two rubber membranes, an electrolyte solution, a sensing unit, a flow channel, a metal frame, a plexiglass shell and fixed base [27]. Two rubber membranes are sealed on both sides of the plexiglass shell and connected by a flow channel to form a sealed cavity, which is filled with the electrolyte solution (a mixed solution of iodine and potassium iodide in which the concentration of potassium iodide is much higher than that of iodine), and the sensing unit is fixed in the middle of the flow channel and immersed in the electrolyte solution. The structure diagram of the sensing unit is shown in Figure 1b, including two anodes, two cathodes and a large number of flow holes.

Like moving-coil seismometers, the electrochemical seismometer is an inertial device. When detecting external vibration, it can be divided into two modules: (1) vibration pickup module, which converts the external vibration into the relative movement of the electrolyte solution and the sensing unit; (2) electromechanical conversion module, with which the sensing unit converts the flow of electrolyte solution into an electrical signal.

The vibration pickup module is composed of two rubber membranes, a metal frame and the electrolyte solution. Two rubber membranes constitute the elastic part, while the metal frame and electrolyte solution form the block of inertial mass. When vibration occurs, the fixed base and sensing unit vibrate with the ground, while the electrolyte solution and metal frame move relative to the sensing unit due to inertia. Thus, the vibration pickup module converts the external vibration into the flow of the electrolyte solution relative to the sensing unit.

**Figure 2:**
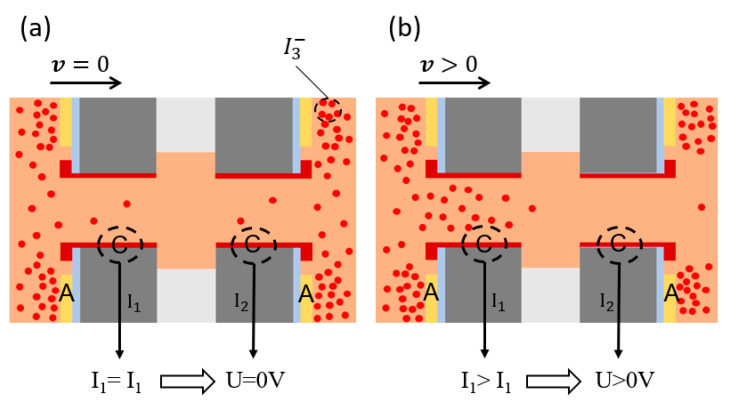
The working principle diagram of electromechanical conversion module, including two states: (**a**) when there is no vibration and (**b**) when there is an external vibration.

The activity of the electromechanical conversion module is accomplished by the sensing unit. Figure 2 illustrates the working principle of the electromechanical conversion module, where the red dots represent the triiodide ions. In the mixed solution of iodine and potassium iodide, there is a complexation process [28]:

(1) $$I_{2}+I^{-}=I_{3}^{-}$$

As the concentration of potassium iodide is much higher than that of iodine, iodine is almost entirely converted to triiodide ions. By applying a DC bias between the anode and cathode, electrochemical reactions occur at the anode and cathode, respectively [28]:

(2) $$\mathrm{Anode}:3I^{-}-2e^{-}\to I_{3}^{-}$$

(3) $$\mathrm{Cathode}:I_{3}^{-}+2e^{-}\to 3I^{-}$$

As the reactions continue, the triiodide ions accumulate near the anode and are depleted near the cathode, while the triiodide ions diffuse from high to low concentrations. Finally, the whole process reaches dynamic equilibrium, and the concentration of triiodide ions is relatively high near the anode and extremely low near the cathode. When there is no vibration, since two pairs of anodes and cathodes of the sensing unit are completely symmetrical, the concentration distribution of triiodide ions near the sensing unit is completely symmetrical, so the output current of two cathodes is equal, and the output voltage is zero (as shown in Figure 2a).

When vibration occurs, the electrolyte solution flows relative to the sensing unit due to inertia. Assuming that the electrolyte solution flows to the right, the concentration of triiodide ions near the left cathode increases, while the concentration of triiodide ions near the right cathode decreases. Correspondingly, the output current of the left cathode increases and the output current of the right cathode decreases (as shown in Figure 2b). After the current/voltage conversion and differential amplification, the final output voltage is given by:

(4) $$U=A(I_{1}-I_{2})R$$

where $I_{1}$ and $I_{2}$ represent the output current of two cathodes, A represents the differential magnification factor and R represents the voltage–current conversion resistance.

In summary, when vibration occurs, the vibration pickup module converts the external vibration into the flow of electrolyte solution relative to the sensing unit, and then the electromechanical conversion module converts the flow of electrolyte solution into the output voltage. In this way, the electrochemical seismometer converts the external vibration into the output voltage.

## 3. Numerical Simulation

**Figure 3:**
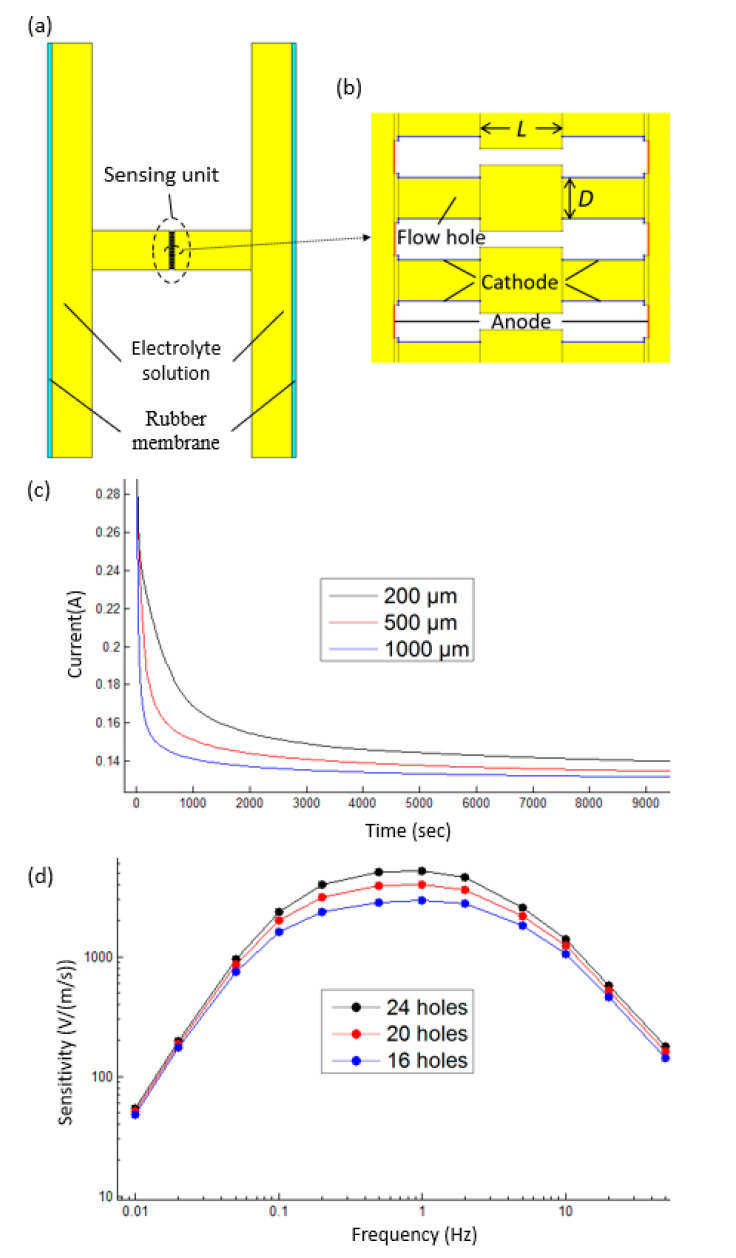
(**a**) Two-dimensional simulation model of the proposed electrochemical seismometer using COMSOL Multiphysics. (**b**) Enlarged view of the sensing unit with four electrodes integrated on one silicon–glass–silicon bonded wafer. (**c**) The output current curves of a single cathode of the electrochemical seismometer with a glass thickness of 200 μm, 500 μm and 1000 μm when there is no vibration. (**d**) Simulation outputs (sensitivity vs. input vibration frequency) of the electrochemical seismometer with 24, 20 and 16 flow holes, showing the relationship between the sensitivity of the electrochemical seismometer and the number of flow holes in the sensing unit.

In order to better study the characteristics of the proposed electrochemical seismometer, finite element simulation (COMSOL Multiphysics, Stockholm, Sweden) was used in this study. Figure 3a presents the two-dimensional simulation model of the proposed electrochemical seismometer, which consisted of two rubber membranes, electrolyte solution and a sensing unit. Figure 3b is the enlarged view of the sensing unit with four electrodes integrated on one silicon–glass–silicon bonded wafer with key geometric parameters as follows: the number of flow holes (*N*), thickness of the glass layer (*L*) and diameter of flow holes (*D* = 80 μm).

In this simulation, the “fluid–structure interaction” physical field was used to convert the initial input volumetric force into the flow velocity in the flow channel, while the coupling physical fields of “laminar flow” and “tertiary current distribution” were used to convert the flow velocity in the flow channel into the output current. In addition, the current-to-voltage conversion resistance was 1 kΩ, and the differential magnification factor was 3.4. Furthermore, the initial concentration of triiodide ions and iodide ions was set to 0.02 mol/ L and 2 mol/L.

Figure 3c presents the time-dependent output current curves of a single cathode of the electrochemical seismometer after the application of the voltage, with thickness of the glass layer (*L*) of 200 μm, 500 μm and 1000 μm, respectively. In addition, the number of flow holes (*N*) in the sensing unit was 24. Obviously, as the thickness of the glass layer (*L*) decreased, the time required for the electrochemical seismometer to reach the steady state decreased rapidly, thus providing an enhanced time-domain stability. The reason is that the volume of the electrolyte solution between two cathodes decreased with the decrease in the thickness of the glass layer (*L*), thus reducing the time required for the electrochemical seismometer to reach the steady state.

Figure 3d shows the simulation results (sensitivity vs. input vibration frequency) of 24-, 20- and 16-flow hole electrochemical seismometers, with the frequency ranging from 0.01 Hz to 50 Hz. In addition, the thickness of the glass layer (*L*) was 200 μm. It can be seen that with the decrease in the number of flow holes in the sensing unit, the sensitivities of the developed electrochemical seismometers at an intermediate frequency decreased significantly, and the sensitivities at low-frequency and high-frequency domains also decreased to an extent, while the 3dB bandwidth increased obviously (see Table 1). This was because the flow resistance of the seismometer increased with the decrease in the number of flow holes (*N*), resulting in the decrease in the sensitivity of the seismometer, while the reduction in the cathode area caused by the decrease in the number of flow holes (*N*) slightly reduced the sensitivity of the detector.

According to the results of numerical simulations and the limitations of actual microfabrication, the geometric parameters of the sensing unit including *L* and *N* were set as 300 μm and 24, respectively.

## 4. Fabrication

**Figure 4:**
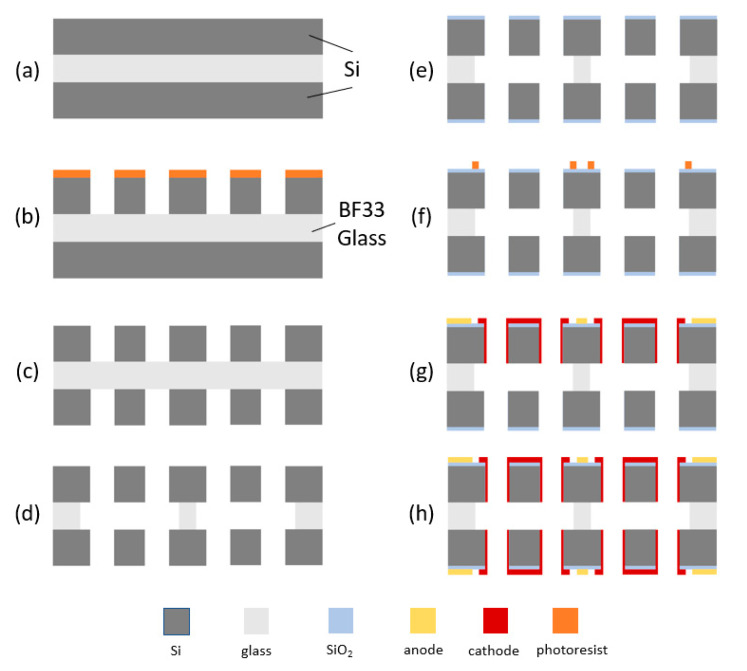
The fabrication processes of the sensing unit with four layers of electrodes integrated on one silicon–glass–silicon bonded wafer: (**a**) preparation of silicon–glass–silicon bonded wafer, (**b**) lithography and deep reactive ion etching (DRIE) on one side, (**c**) lithography and DRIE on the other side, (**d**) etching the glass layer to form flow holes, (**e**) chemical vapor deposition (CVD) SiO_2_ on both sides, (**f**) lithography on one side, (**g**) sputtering and patterning anode and cathode and (**h**) lithography, sputtering and patterning anode and cathode on the other side.

Figure 4a–h shows the fabrication process of the sensing unit with four electrodes integrated on one silicon–glass–silicon bonded wafer:

- (a) A silicon wafer and a BF33 glass wafer (Beijing GIN KOO MEMS Scientific & Technological, Beijing, China) were bonded together by anodic bonding, under a voltage of 400 V, a temperature of 350 °C and a pressure of 300 mbar. By maintaining conditions of pressure/temperature, and reversing voltage, another silicon wafer was bonded to the other side of the glass wafer by anodic bonding. In this paper, the thickness of the two silicon wafers was 200 μm, and the thickness of the glass wafer was 300 μm.
- (b) Photoresist AZ4620 (AZ Electronics Materials, Somerville, USA) was spin-coated on the front of the silicon–glass–silicon bonded wafer, followed by exposure and development (developer: 0.6% NaOH) to form a patterned photoresist. Next, using the patterned photoresist as a mask, the silicon layer of the wafer was completely etched by deep reactive ion etching (DRIE). Finally, the remaining photoresist was removed by acetone and then the wafer was thoroughly rinsed.
- (c) As in step (b), the silicon layer on the other side of the silicon–glass–silicon bonded wafer was etched completely by DRIE.
- (d) The intermediate glass layer was etched from both sides of the silicon–glass–silicon bonded wafer by the vapor of hydrofluoric acid to form flow holes. Then, the wafer was cleaned with heated concentrated sulfuric acid. In addition, since the hydrofluoric acid etched the glass isotropically, there were some lateral erosions in the intermediate glass layer.
- (e) SiO_2_ (0.6 μm in thickness) was deposited on both sides of the silicon–glass–silicon bonded wafer and the side wall of the flow holes by chemical vapor deposition (CVD) to form insulating layers of anodes and cathodes.
- (f) A dry film of photoresist SD230 was pasted on the front side of the silicon–glass–silicon bonded wafer, and then exposed and developed (developer: 0.85% Na_2_CO_3_). The residual photoresist was then removed by oxygen plasma (2 min).
- (g) Titanium (Ti) (40 nm in thickness) and platinum (Pt) (200 nm in thickness) were sputtered on the patterned dry-film photoresist in sequence. Then, the patterned anodes and cathodes were formed by metal lift-off technology, followed by the addition of sodium hydroxide solution or acetone to remove residual photoresist.
- (h) As in step (f–g), the patterned anodes and cathodes on the other side of the silicon–glass–silicon bonded wafer were formed in the same way.

As shown in Figure 4f–h, the lateral erosions of the intermediate glass layer ensured the effective insulation of two cathodes. In addition, electrodes were connected and led out by leading wires fabricated on the wafer.

**Figure 5:**
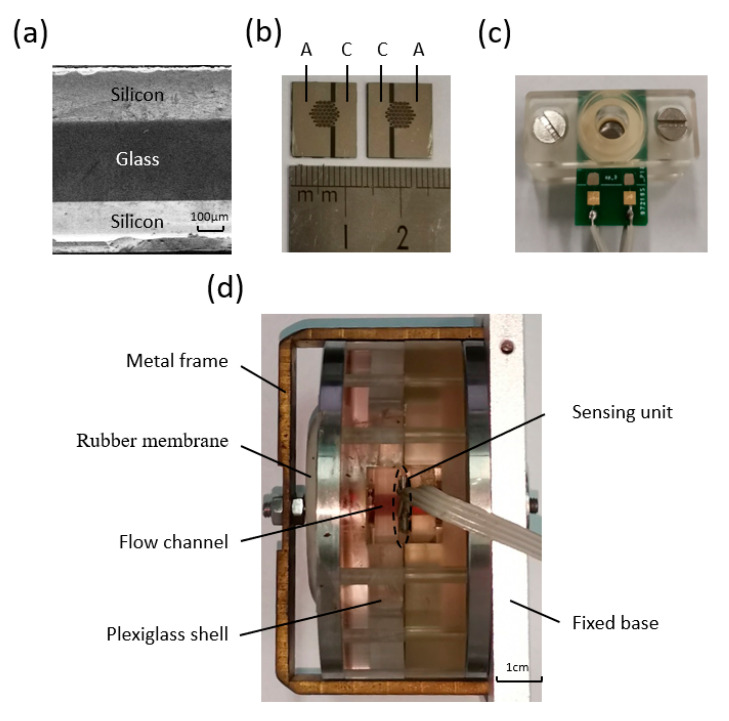
(**a**) Side view of the sensing unit, and assembly process of the electrochemical seismometer (**b**–**d**), including (**a**) both sides of the sensing unit, (**c**) the assembled sensing unit and (**d**) the electrochemical seismometer.

Figure 5a shows a scanning electron microscope (SEM) picture of the silicon–glass–silicon bonded wafer, indicating high quality of the two bonding interfaces. Figure 5b shows the fabricated sensing unit with two completely symmetrical sides. Figure 5c,d show the assembled sensing unit and the electrochemical seismometer.

In this study, all the fabrication processes were easy to implement. Due to the completely symmetrical processes of the two sides, the fabricated sensing unit had high symmetry. Benefiting from alignments of lithography, the alignment accuracy of the four electrodes was greatly improved at a sub-micron level, and the consistency of the sensing unit was greatly improved. Since the sensing unit only needed one chip, this dramatically reduced the difficulty of device assembly.

## 5. Device Characterization

In order to demonstrate the performance of the fabricated electrochemical seismometers, this paper tested the sensitivities of four devices based on well-defined inputting signals as a function of frequency and amplitude. Moreover, the test results in response to random ground motions of the fabricated electrochemical seismometer were compared with the commercially available electrochemical seismometer CME6011 (R-sensors, Moscow, Russia).

### 5.1. Sensitivity Characterization

The sensitivity of the electrochemical seismometers was calibrated on an ultra-low-frequency vibration table (National Institute of Metrology of China). The vibration table applied vibrations of 20 frequencies ranging from 0.01 Hz to 50 Hz, then the output voltages of the electrochemical seismometers were measured to calculate the sensitivity of the electrochemical seismometers at different frequencies. In this study, a voltage of 0.3 V was applied to anodes, while cathodes were virtually grounded. In addition, the current-to-voltage conversion resistance was 1 kΩ, and the differential magnification factor was 3.4. Furthermore, the electrolyte solution was a mixture of 0.02 mol/L iodine and 2 mol/L potassium iodide.

**Figure 6:**
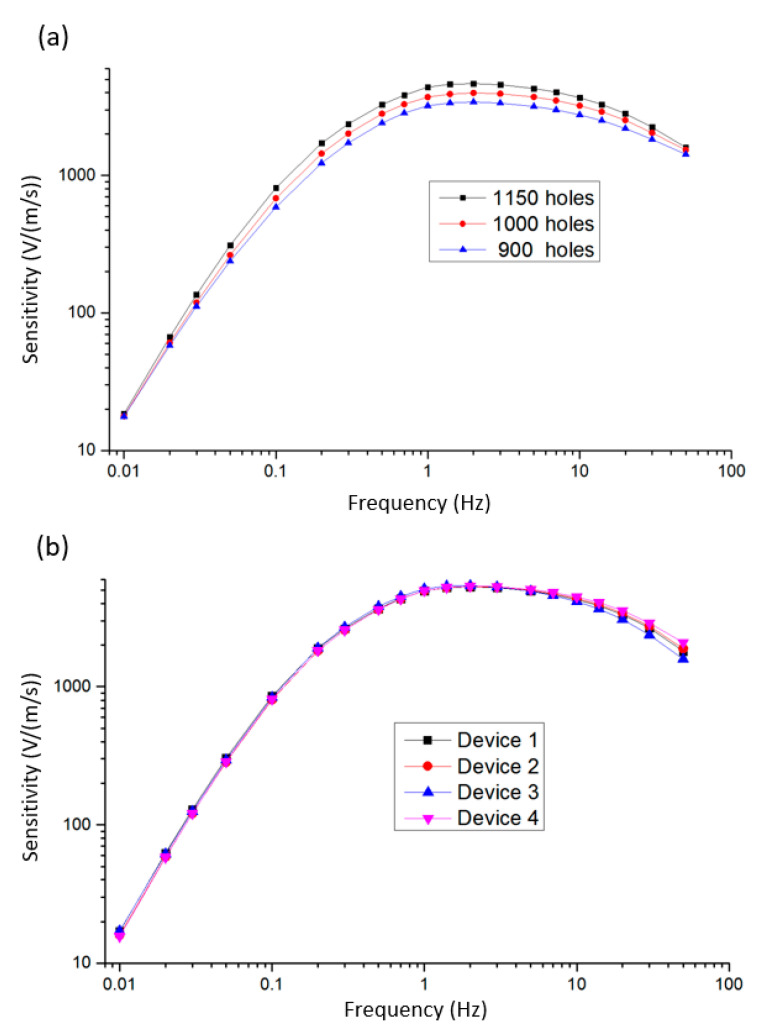
(**a**) The test results (sensitivity vs. input vibration frequency) of the fabricated electrochemical seismometers with 1150, 1000 and 900 flow holes, respectively. (**b**) The sensitivity curves with mean and standard deviation of four devices with 1150 flow holes.

Figure 6a shows the test results (sensitivity vs. frequency) of the fabricated electrochemical seismometers with 1150, 1000 and 900 flow holes, respectively. It can be seen from the experimental results that with the decrease in the number of flow holes, the sensitivities of the fabricated electrochemical seismometer decreased and the bandwidth increased (see Table 2). Compared with the simulation results, it can be confirmed that the sensitivity is negatively correlated with the number of flow holes, while the bandwidth is positively correlated with the number of flow holes.

Figure 6b shows the sensitivity curves of four devices with 1150 flow holes, which were quantified as 827.15 ± 23.74 V/(m/s) at 0.1 Hz, 4997.92 ± 77.97 V/(m/s) at 1 Hz and 4310.66 ± 143.70 V/(m/s) at 10 Hz with the peak sensitivity of 5345.45 ± 43.78 V/(m/s) at 2 Hz. The experimental results showed high consistency of the developed electrochemical seismometer, which can be explained by the consistency of the sensing units. Benefitting from the extreme symmetry of the two sides, as well as the high alignment of the four electrodes and the upper and lower flow holes, the consistency of the sensing units was greatly improved. The alignment accuracy of the upper and lower flow holes affect the flow resistance, and therefore affect the sensitivity curve of the device. Therefore, the higher alignment accuracy significantly improved the consistency of the electrochemical seismometer.

### 5.2. Response to Random Ground Motions

In order to further evaluate the performances of the developed electrochemical seismometer, the electrochemical seismometer developed in this study and the commercial counterpart of CME6011 were positioned on the same laboratory floor to monitor random vibrations. To minimize impacts of external noises, devices were tested late at night in a quiet environment.

**Figure 7:**
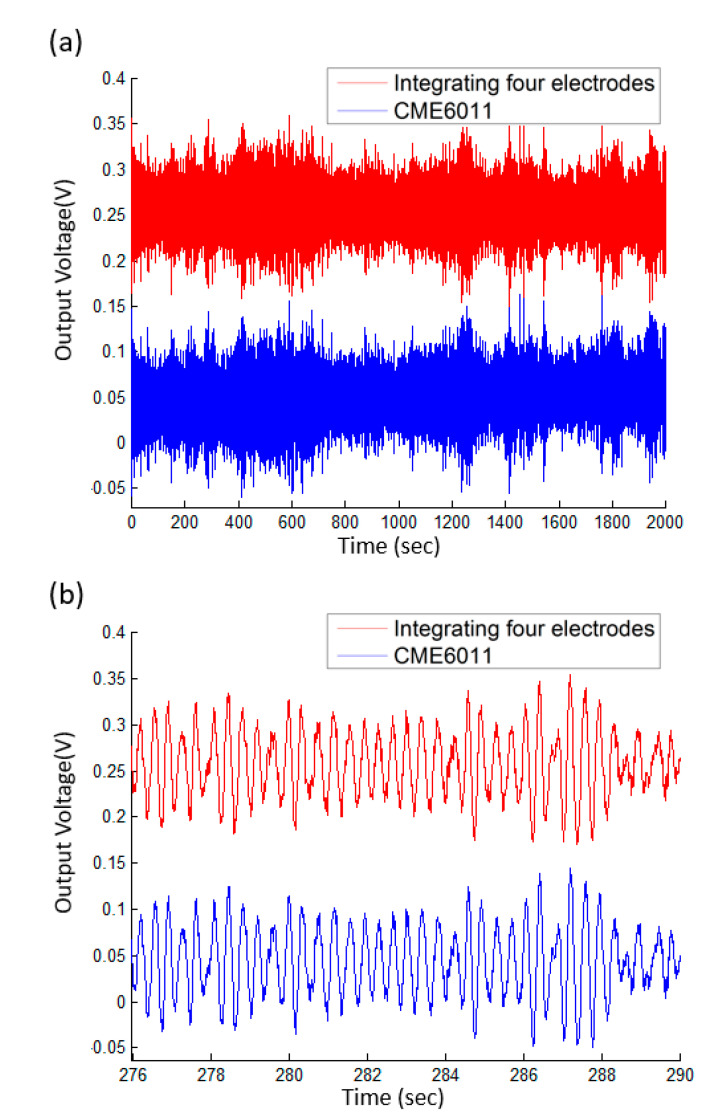
Test results of the electrochemical seismometer developed in this study in comparison with the commercial counterpart of CME6011 without observable ground motions, including (**a**) the normalized time-domain response and (**b**) enlarged view of the time-domain response from 276 to 290 s.

Figure 7a shows the time-domain response of the fabricated electrochemical seismometer and the reference of CME6011 whose output voltage was amplified and shifted. Figure 7b shows the enlarged view of the time-domain response to microvibrations from 276 to 290 s, where a high consistency between the developed electrochemical seismometer and the reference of CME6011 was found. The corresponding correlation coefficient was quantified as 0.966, which validated the performance of the developed electrochemical seismometer.

**Figure 8:**
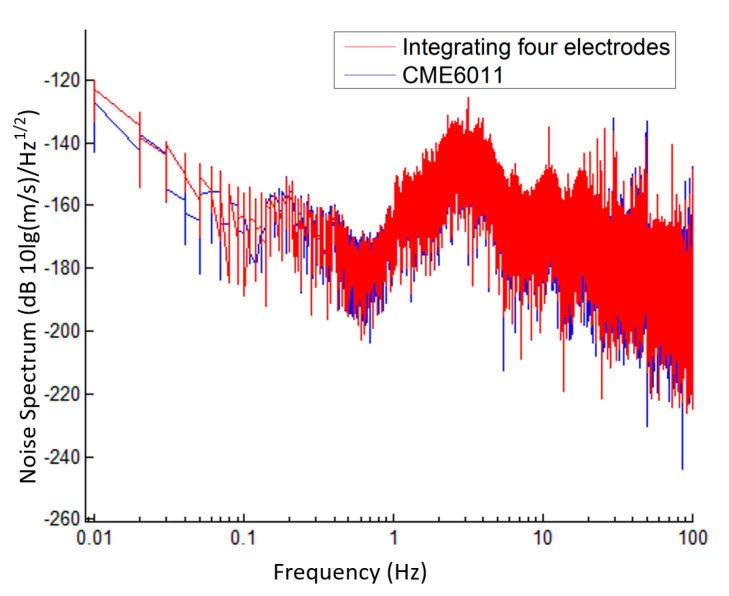
The noise spectrum density results of the fabricated electrochemical seismometer with four electrodes integrated on one chip in comparison with the commercial counterpart of CME6011.

Figure 8 shows the noise spectrum of the fabricated electrochemical seismometer with four electrodes integrated on one chip in comparison with those of the reference of CME6011, which were quantified as −163.49 vs. −167.09 dB at 0.1 Hz, −168.76 vs. −168.00 dB at 1 Hz and −160.33 vs. −159.51dB at 10 Hz. As shown in the figure, the developed electrochemical seismometer and the reference of CME6011 demonstrated high consistencies in the frequency domain of 0.1–100 Hz. When the time-domain consistency was also taken into consideration, it was concluded that the self-noise level of the developed electrochemical seismometer and the reference of CME6011 was much lower than the noise levels of ground vibrations, which further validated the low-noise performance of the developed electrochemical seismometer.

## 6. Conclusions

This paper introduced a MEMS-based electrochemical seismometer with four electrodes integrated in a sensing unit. The new manufacturing method of the sensing unit improved the alignment accuracy of the four electrodes to the sub-micron level, which greatly improved the consistency of the sensing units and then electrochemical seismometer. The simulation results showed that the time required for the developed electrochemical seismometer to reach the steady state became shorter with the decreasing thickness of the glass layer, which proved that the developed electrochemical seismometer with a thin glass layer had excellent time-domain stabilities. The sensitivity test results showed that the developed electrochemical seismometer had the characteristics of high sensitivities and high consistencies among individual devices. Compared with the reference of CME6011, the developed electrochemical seismometer showed comparable noise levels. These results fully illustrated the performance of the electrochemical seismometer developed in this study with a great potential in various applications.

## Figures and Tables

**Table 1:** Performance comparisons of electrochemical seismometers with 24, 20 and 16 flow holes.

Performance	24 Holes	20 Holes	16 Holes
Maximum sensitivity	4967.5 V/(m/s) ≅1 Hz	3842.9 V/(m/s) ≅1 Hz	2844.7 V/(m/s) ≅1 Hz
3 dB bandwidth	0.18–2.80 Hz (1.19dec)	0.18–2.80 Hz (1.26dec)	0.18–2.80 Hz (1.36dec)

**Table 2:** Performance comparisons of electrochemical seismometers with 24, 20 and 16 flow holes.

Performance	1150 Holes	1000 Holes	900 Holes
Maximum sensitivity	5345.45 V/(m/s) ≅2 Hz	3987.14 V/(m/s) ≅2 Hz	2844.7 V/(m/s) ≅2 Hz
3 dB bandwidth	0.51–13.86 Hz (1.43dec)	0.50–15.07 Hz (1.48dec)	0.50–15.34 Hz (1.49dec)
